# Exploring the factors affecting elementary mathematics teachers’ innovative behavior: an integration of social cognitive theory

**DOI:** 10.1038/s41598-024-52604-4

**Published:** 2024-01-24

**Authors:** Kai Li, Tommy Tanu Wijaya, Xiaoying Chen, Muhammad Syahril Harahap

**Affiliations:** 1 Teacher Education Collage, Chongqing University of Education, Chongqing, China; 2 School of Mathematical Sciences, Beijing Normal University, Beijing, China; 3 Institut Pendidikan Tapanuli Selatan, Padangsidimpuan, Indonesia; 4 Education Bureau of Jinwan District, Zhuhai, China

**Keywords:** Psychology, Human behaviour

## Abstract

Teacher innovative behavior is one of the vital factors, affecting student engagement, addresses diverse needs, promotes critical thinking, fosters lifelong learning, and contributes to educational research and development. By encouraging and supporting teacher innovation, we may can ensure that education remains relevant, effective, and impactful in preparing students for the future. Teacher innovative behavior is also needed to improve the mathematics skills of elementary school students, and it is important to determine the predictors that significantly affecting Teacher innovative behavior. Therefore, this study aimed to develop a model that predicted possible factors affecting mathematics teachers' innovative behavior based on Social Cognitive Theory (SCT). Data were collected from 132 elementary school teachers in China to verify the model, and the analysis was conducted using a structural equation modelling approach. Theoretically, 10 of the 15 hypotheses were found to be significant. The results showed that facilitating conditions and self-efficacy significantly affect mathematics teachers' innovative behavior. Meanwhile, Technological, Pedagogical and Content Knowledge (TPACK) knowledge, Social Influences, Rewards, Work engagement and anxiety did not show any effect. The contribution developed a model and provided new knowledge about the factors affecting elementary school teachers' innovative behavior. Practically, this could be used to improve teachers' innovative behavior.

## Introduction

Teacher innovative behavior is crucial for the sustainability of education systems and the overall development of students. In today's rapidly changing world, where new technologies, pedagogical approaches, and societal needs emerge, teachers need to adapt and innovate to meet the evolving demands of education. Enhancing innovative behavior has emerged as a significant area of focus in the twenty-first century^1^. This behavior is widely acknowledged to yield positive outcomes, benefiting both teacher performance during instruction and student capabilities^2,3^. As Docherty^4^ argued, the introduction of teacher innovative behavior can greatly optimize the learning process, fostering an environment that is conducive to heightened student engagement. Furthermore, scholarly literatures indicates that embracing innovative behavior empowers teachers to stay informed about the evolving teaching challenges within the dynamic educational landscape^5,6^.

Teachers' innovative behavior encompasses the generation of creative ideas to revolutionize teaching styles and instructional models^7,8^. The current Chinese government has issued many new goals that focus on the ability to innovate and foster this concept^9,10^. Many studies show that the use of various kinds of technology-based learning tools^11–14^, innovative learning models^15,16^, STEM education^17–19^ and other innovations continue to increase. The innovation ability of teachers may not maximal and their behavior still needs attention and improvement. Therefore, different studies should be carried out to encourage innovative behavior in mathematics teachers.

This innovative behavior may be more difficult to improve due to neoliberal reasons, and the strong effects of standardization. Mathematics teachers encounter two primary challenges that impede their ability to foster creativity and novelty in the design of teaching and learning activities. Firstly, they often rely on established teaching habits and methods that hinder their willingness to explore alternative approaches. Secondly, some perceive themselves as lacking inherent creativity, further inhibiting their confidence in innovative practices. This study shows instances where teachers incorporate innovations into instructional activities. However, these innovations are often dictated by administrative obligations and school standards rather than self-generated creative endeavors. Several barriers such as the standardization of teaching and learning activities focused on individual students' mathematical abilities and learning outcomes, leading to a decrease in innovation.

Given the following context, it is crucial to identify the factors that influence innovative behavior and explore ways to enhance the innovative behavior of elementary mathematics teachers. Previous results established that Social Cognitive Theory (SCT) encompasses internal and external factors impacting individual behavior. Numerous studies have used SCT to construct models for comprehending individual behavior^20,21^. In alignment with previous results, this study employs the concept to investigate the environmental and internal factors that potentially influence the innovative behavior of mathematics teachers. The initial hypothesis is examined and analyzed utilizing the Partial Least Squares Structural Equation Modeling (PLS-SEM) technique.

The findings are useful for closing the study gap regarding factors increasing teachers’ innovative behavior. This study focused on answering the question of which predictors significantly affect teacher innovative behavior, especially at the elementary school level under the Social Cognitive Theory. The strongest predictor that influences teacher innovative behavior, especially at the elementary school level, is the level of support and encouragement received from school leadership and administrators. The findings are expected to contribute both practically and theoretically to teachers, and school principals.

## Literature review

### Mathematics teachers' innovative behavior

Innovative behavior is defined as the creation or innovation conducted to improve performance in the work environment^22,23^. According to Hunter^24^ in the context of mathematics teaching, there are 3 main indicators for measuring innovative behavior. First, developing an innovative learning environment that benefits all students and the second indicator is innovative tasks, supporting pedagogical practices. The third is the use of new learning media, aligning with mathematics teaching activities. According to Wei et al.^25^ there were 5 main indicators of teaching innovation in mathematics classes, namely interactive discussions, open-ended activities, mathematics problem-solving, multilevel teaching, and independent teaching. In conclusion, innovative behavior is the innovative ideas of mathematics teachers at the elementary school level to innovate with their teaching styles and models to improve student mathematics outcomes.

Several studies succeeded in proving that Innovative behavior is one of the significant components of teacher-teaching success^26,27^. Therefore, Innovative behavior plays an important role in improving student performance and school progress which is the concern of a mathematics teacher^28,29^. However, this study shows that teachers' innovative behavior is still low and needs attention^2,30^. Therefore, studies are needed to theoretically, practically, and significantly increase teachers’ innovative behavior.

In the context of education, the teaching approaches employed by teachers exhibit significant flexibility and adaptability in response to the diverse circumstances and conditions encountered in the classroom^31,32^. Teachers possess the capacity to innovate by incorporating various learning models and media to effectively accomplish the objectives of mathematics education. However, they may be inclined to maintain a risk-averse mindset, hesitant to adopt new teaching methods that might not yield immediate success in enhancing teaching performance. According to Bandura^33^, the process of innovation is beset with challenges, gradual in nature, yields unpredictable outcomes, and entails relatively low success rates. These factors constitute the underpinnings for the low nature of teacher innovative behavior. The Chinese government persists in its dedication to discerning the determinants capable of exerting an impact on the variable. Subsequently, appropriate policies and programs will be designed to enhance teacher innovative behavior.

The Chinese government places a strong emphasis on innovation and creativity^34^. It recognizes the significance of innovation in the education world and states that China needs to cultivate a culture of innovation. Furthermore, there is no institution more effective at fostering innovation and creativity than schools. The Ministry of Education (MOE) has also issued numerous policies to support teaching innovation^35^. Based on this background, this study shows that the determinants influencing the innovative behavior exhibited by teachers within educational institutions are significant.

Furthermore, mathematics at the elementary school level is an important stage that focuses on basic knowledge, affecting students' abilities at the secondary school level. In the new curriculum issued in 2022, China divides mathematics material into algebra, geometry, statistics and mathematical applications in daily life. The government also emphasizes the objectives of teaching and learning the subject at the elementary school level on Knowledge and skills, mathematical thinking, problem-solving, and emotional attitudes. Subsequently, the four aspects are divided into more detailed learning activity objectives. At the elementary school level, mathematics material is quite complex and teachers need innovative ideas to teach effectively. Innovative behavior may be important in increasing student creativity, problem-solving skills, and critical thinking^8^. Considering the factors affecting mathematics, the concept may accelerate and encourage innovative behavior of a mathematics teacher appropriate to the learning objectives in the learning curriculum issued by the Chinese government.

### Social cognitive theory

To overcome the issues related to teachers’ innovative behavior, Social Cognitive Theory (SCT) is one of the theories used to analyze the factors influencing individual behavior. SCT, as elaborated by Bandura^36^ explained that individual behavior was affected by two primary factors, namely internal and environmental. This theory has been widely used and applied in various fields, particularly in education^37–39^. Previous studies suggested the need to develop and modify environmental and internal factors related to innovative behavior^20^. In terms of environmental factors, social influences and facilitating conditions were explored in previous studies. For individual internal factors, technology literacy, stress, and individual engagement are associated with innovative behavior. Based on the context, this study divides environmental factors into facilitating conditions, social influences and rewards appropriate to predictors that are often used in previous results.

Social influences in the context of this studies are defined as people around elementary mathematics teachers who believe that innovative behavior can improve teaching performance and positively affect students. Wu^20^ found that Social influences is a vital predictor of innovative teaching in China The role of teachers is to continuously learn and develop their teaching skills. In the twenty-first century, TPACK knowledge, proposed by Mishra^40^ is believed to be a comprehensive framework, guiding teachers in teaching and serving as a foundation for instructional innovation. The support from people around teachers can enhance their enthusiasm to continue learning and mastering the Technological Pedagogical Mathematical Knowledge (TPMK) ability. Additionally, engagement has been proven to be positively affected by social influence^41^. Engagement among elementary school teachers is likely to improve significantly when enhanced support is received from their peers and colleagues. Having a strong team and support network can foster an environment conducive to innovation in their teaching practices. The assistance and encouragement may also lead to increased recognition and emotional support, which can play a vital role in motivating teachers to persevere and continue their innovative efforts within the school setting. Guo^42^ substantiated the powerful impact of social support in effectively reducing individuals' anxiety levels. Therefore, when teachers embark on innovative approaches, their primary concern often revolves around the fear of potential negative consequences on students' learning outcomes. Social influence can reduce the anxiety of elementary school teachers in innovating their teaching practices. Kuriawan^5^ emphasized that support, direction, and feedback from people and the environment are needed to improve teachers' innovative behavior. The initial hypothesis is formulated as follows:

#### H1

Social influence has a positive effect on the TPACK ability mathematics teachers at elementary school mathematics teachers.

#### H2

Social influence positively affects the work engagement of mathematics teachers at the elementary school level.

#### H3

Social influence positively affects the self-efficacy of mathematics teachers at the elementary school level.

#### H4

Social influence has a negative effect on the anxiety of mathematics teachers at the elementary school level.

#### H5

Social influence has a positive effect on mathematics teachers' innovative behavior at the elementary school level.

Furthermore, elementary mathematics teachers' innovative behavior may be affected by the rewards obtained by teachers. Moreover, rewards are always believed to work to improve individual performance and behavior^43^, including in the context of education. Teachers may be motivated to seek rewards, significantly affecting work engagement^44^. Moreover, teachers may feel valued when they successfully innovate in classroom practices, especially when their innovations lead to improved student learning outcomes. With rewards given to teachers for their innovations, the anxiety associated with innovation among teachers may decrease. The previous result predicted that rewards are related to individual behavior^45,46^. Therefore, the reward factor may be able to encourage mathematics teachers to innovative behavior. Based on the literature review, the initial hypothesis is that:

#### H6

Rewards have a positive effect on the TPACK ability of mathematics teachers at the elementary school level.

#### H7

Rewards have a positive effect on the work engagement of mathematics teachers at the elementary school level.

#### H8

Rewards have a positive effect on mathematics teacher self-efficacy at the elementary school level.

#### H9

Rewards have a negative effect on the anxiety of mathematics teachers at the elementary school level.

#### H10

Rewards positively affect mathematics teachers' innovative behavior at the elementary school level.

The last Factor environmental is Facilitating conditions. FC are predicted as the main key and directly affect mathematics teachers' innovative behavior. A teacher can innovate in teaching and learning activities with supportive school facilities. Wijaya^47^ found that Facilitating conditions is the significant factor affecting mathematics Teachers' Behavior. Based on the literature review, the initial hypothesis is that:

#### H11

Facilitating conditions have a positive effect on mathematics teachers' innovative behavior at the elementary school level.

Regarding internal factors, TPACK ability, work engagement, self-efficacy, and anxiety are believed to affect mathematics teachers' innovative behavior. TPACK ability was first introduced by Shulman^48^, and mathematics teachers need technological, pedagogical and strong mathematical knowledge before innovating in learning. Teachers should be able to combine learning models and technology-based media^49^, specifically on algebra and geometry problems. This is predicted to have a strong relationship with mathematics teachers' innovative behavior in teaching and learning activities. Based on the literature review, the initial hypothesis is that:

#### H12

TPACK ability has a positive effect on mathematics teachers' innovative behavior at the elementary school level.

Previous study has widely used work engagement to analyze professionalism and performance in teaching^1,4,50^. The concept can be interpreted as the individual level of seriousness to give effort in work matters. Several studies have proven that teacher work engagement is a significant predictor affecting job performance, job satisfaction, and commitment, as well as increasing creativity and innovation in teaching methods affecting teaching performance^1^. Based on the literature review, the initial hypothesis is that:

#### H13

Work engagement has a positive effect on mathematics teachers' innovative behavior at the elementary school level.

**Self-efficacy** refers to teachers' personal beliefs in the ability to effectively perform behaviors that contribute to the improvement of their teaching performance. It has been widely used in the educational context in previous studies related to the behavior of a teacher or student^8^. The concept significantly affects mathematics teachers' innovative behavior ^3,8^. Based on the literature review, the initial hypothesis is that:

#### H14

Self-efficacy has a positive effect on mathematics teachers' innovative behavior at the elementary school level.

In the context of this study, **anxiety** is defined as the tendency of teachers to be uneasy and nervous about innovating by teaching mathematics at the elementary school level. Many studies support that anxiety has a negative effect on a person's innovation^51–53^. The many tasks and amnesty of the school and the fear of their innovations failing to improve students' mathematics ability may have a significant negative effect on innovative behavior. Based on the literature review, the initial hypothesis is that:

#### H15

Anxiety has a negative effect on mathematics teachers' innovative behavior at the elementary school level.

**Figure 1:**
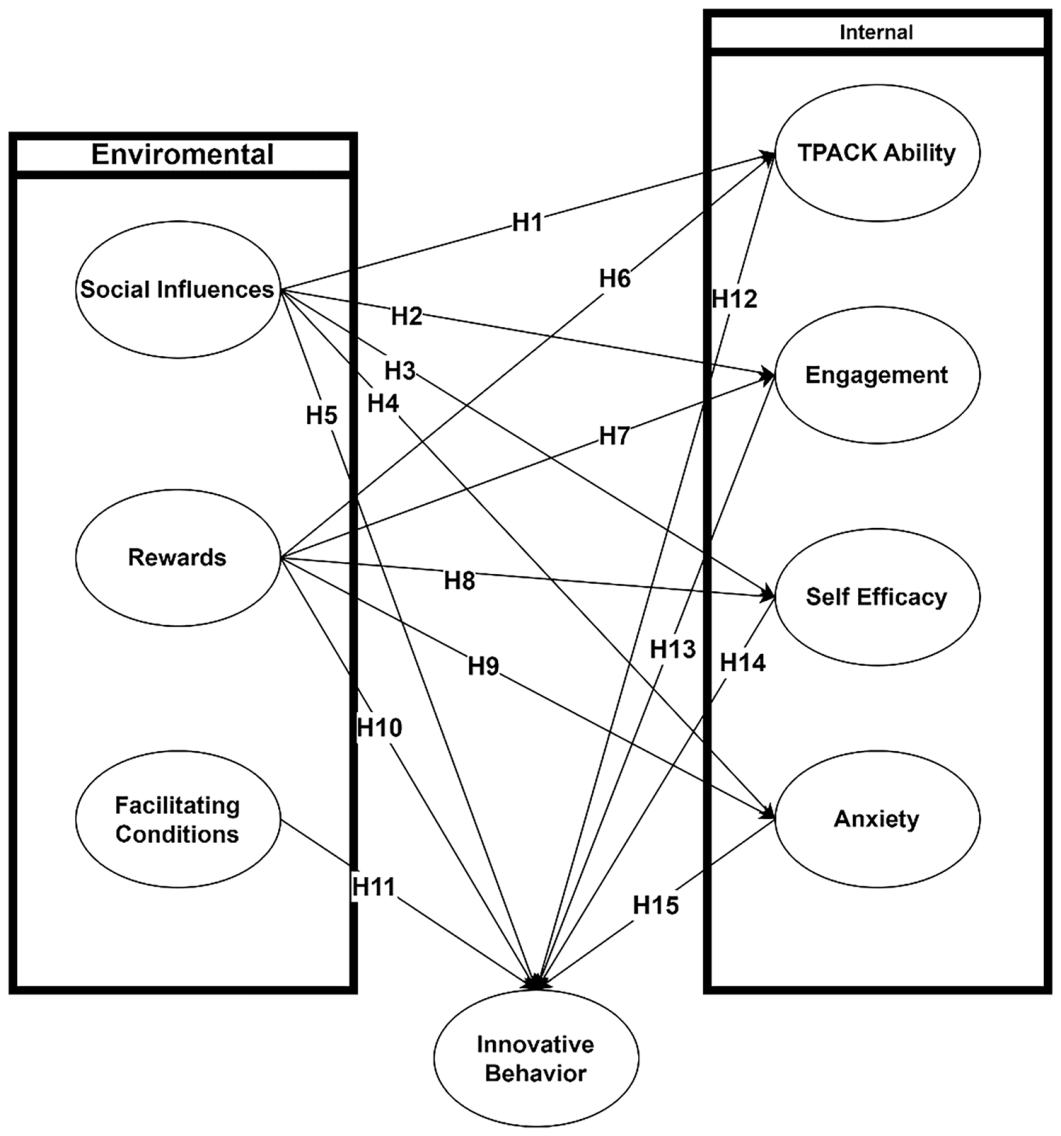
Framework model.

Based on the description of the literature review, predictors affecting elementary mathematics teachers innovative behavior consist of seven independent, four intermediate and one dependent variable, resulting in 15 initial hypotheses, as seen in Fig. 1.

## Methodology

This study determines the factors affecting mathematics teachers' innovative behavior at the elementary school level. To achieve this goal, quantitative methods are used by distributing questionnaires and processing the data with PLS-SEM techniques for hypothesis testing.

### Participants

This study collected questionnaire data from 132 elementary mathematics teachers on the factors that affect teachers' innovative behavior. About 61.36% and 38.64% of participants were female and male elementary mathematics teachers. From the study, 75.76% of elementary mathematics teachers had an undergraduate education level while 24.24% had a graduate education level. Furthermore, 57.58% of school locations are in rural areas and 42.42% are in urban areas. The respondents in this study, who have been part of the teaching experiment, are distributed in a balanced manner. About 34.85% possess teaching experiences of under five years, while 33.33% have accumulated 6–15 years of teaching experience. Additionally, 31.81% boast a considerable teaching experience of more than 15 years, and Table 1 shows the main demographic respondents. The structural equations model sample size was better if not less than 100^54^. Therefore, this study reached the recommended sample respondent provisions.

**Table 1:** Respondent demographic data.

Demographic	Type	N	Percentage
Gender	Male	51	38.64
	Female	81	61.36
Level of education	Undergraduate	100	75.76
	Graduate	32	24.24
School location	Urban	56	42.42
	Rural	76	57.58
Teaching experiences	Less than 5 years	46	34.85
	between 6 and 15 years	44	33.33
	Over 15 years	42	31.81
Total		132	100

### Instrument and data collection

The entire questionnaire used was adopted from a previous study and supported by a strong literature review (see appendix). The instrument was checked and validated by 2 doctoral students and 1 post-doctoral expert in innovative behavior. This study used the Social Cognitive Theory as the basis for developing all the items in this instrument. The questionnaire was divided into two parts. The first part contained sociodemographics (gender, level of education, school location, teaching experiences), while the second consisted of 22 questionnaires derived from 8 constructs. It was designed with a 5-point Likert scale from 1 = strongly disagree to 5 = strongly agree. First, two postdoctoral fellows designed and modified the instrument taken from previous study. Subsequently, the initial questionnaire was given to 2 professors in the field of mathematics and psychology education. A total of 5 Chinese elementary mathematics teachers were involved in filling out and reviewing the questionnaire to ensure the questionnaire was understandable.

The population in this study were elementary school mathematics teachers from Sichuan province. This study randomly selected 150 schools and administered an online questionnaire. Online questionnaires were considered more effective and efficient for elementary mathematics teachers in China. The utilization ensured that the work time of elementary mathematics teachers remained uninterrupted. These questionnaires were conveniently filled out by the teachers to accommodate their schedules accordingly. Moreover, the implementation facilitated a more comprehensive data collection process, as they were effortlessly disseminated through various platforms and social media channels. The confidentiality of the questionnaire responses was strictly maintained, with the data solely used for study purposes. Human Ethics Approval for the interviews was obtained from the Chongqing University of Education Human Ethics Committee on the 2 February 2023 (Approval number: 202302024). All of the procedures were performed in accordance with the Declaration of Helsinki and relevant policies in China. Before their participation, All participants agreed to participate voluntarily, with informed consent when they filled in the survey and were able to withdraw from the study freely at any time.the distribution of the questionnaires took place between March and May 2023. Ultimately, valid data were collected from 132 elementary mathematics teachers. The data were confidential and participation was anonymous with- out any potential risk to the integrity of the subjects.

### Data analysis

Data analysis used SPSS and SMART-PLS3. SPSS software is used for descriptive statistics data processing, which is a key step in the initial process and data screening, specifically in quantitative study. The second step, SMART-PLS 3 is the main software in PLS-SEM (variance-based SEM) analysis often use to design new study models^55–57^. This study uses PLS_SEM instead of CB_SEM because it is more practical where there is no need to determine the normality of the data^58^. It can also analyze study models with relatively small samples, including many indicators and path relationships^59^. Furthermore, PLS-SEM is more flexible for identifying the relationship between measurement items and each construct compared to CB-SEM^60,61^.

PLS-SEM is a nonparametric algorithm computation used to determine the value of each latent variable^62^. The analysis steps are to enter data information, measure the construct, analyze discriminant validity and determine each relationship between construct variables^63,64^. In study with the PLS-SEM approach, Hair et al.^65^ recommended paying attention to several factors. Analyzing the significance level should be below 0.05 since the relationship between variables is declared significant. The model has good enough explanatory power when R^2^ values are not less than 0.25.

In Partial Least Squares Structural Equation Modeling (PLS-SEM), the evaluation of measurement and structural models follows specific criteria to guarantee the credibility and accuracy of the model.

To begin, the measurement model undergoes rigorous scrutiny. This includes assessing the reliability of the measurement scales, typically done using metrics such as composite reliability (CR) and Cronbach's alpha. Furthermore, the convergent validity of indicators is examined through the average variance extracted (AVE) and factor loadings. An AVE above 0.5 indicates that the observed variables adequately represent the latent construct. Discriminant validity is then confirmed by comparing the square root of AVE with inter-construct correlations, ensuring that different constructs are distinct from one another.

Moving to the evaluation of structural models, path coefficients, indicating the strength and direction of relationships between latent constructs, are scrutinized. Bootstrapping techniques aid in estimating the significance of these coefficients. Examining effect sizes, such as R^2^ values, clarifies the proportion of variance explained in endogenous constructs by their exogenous counterparts.

## Results

### Measurement model evaluation

In PLS-SEM, validity and reliability tests on each construct are verified using the CFA technique^66,67^. As seen in Table 2, all item loadings exceed the minimum criterion of 0.7, hence the construct has a good agreement. The CR value should be more than the 0.7 limits since each construct has good inner consistency^68^. In this study, the CR value ranges from 0.897 to 0.944, indicating the absence of a problem with inner consistency. Furthermore, the Ave value should be above the 0.5 thresholds for the construct to have good convergent validity^68^. The lowest AVE value is 0.744 and it is considered to have reached the minimum criteria. Finally, the Cronbach alpha ranged from 0.807 to 0.896, exceeding the 0.6 threshold recommended by Hair^68^.

**Table 2:** Results for reliability, convergent validity, and multicollinearity test.

Construct	Indicator	Outer loadings	Cronbach's Alpha	Composite reliability	Average variance extracted (AVE)	VIF
Anxiety	AN1	0.913	0.896	0.935	0.827	2.111
	AN2	0.910				2.278
	AN3	0.906				2.140
Work engagement	EN1	0.894	0.867	0.919	0.791	1.606
	EN2	0.898				1.963
	EN3	0.876				1.846
Facilitating conditions	FC1	0.866	0.870	0.920	0.794	2.032
	FC2	0.928				2.477
	FC3	0.878				2.360
Innovative behavior	IB1	0.947	0.882	0.944	0.895	2.386
	IB2	0.945				1.844
Reward	RW1	0.863	0.835	0.901	0.752	1.844
	RW2	0.856				2.366
	RW3	0.881				2.374
Self-efficacy	SE1	0.903	0.885	0.929	0.813	2.300
	SE2	0.889				2.740
	SE3	0.913				2.758
Social influence	SI1	0.893	0.827	0.897	0.744	2.816
	SI2	0.888				2.581
	SI3	0.804				2.649
TPACK	TPACK1	0.901	0.878	0.925	0.804	2.649
	TPACK2	0.891				3.027
	TPACK3	0.897				2.320

### Common method bias

According to the recommendation of Kock^69^, a collinearity test was required in PLS-SEM to determine when the data collected had bias problems. A multicollinearity test was carried out by analyzing the variable inflation factor (VIF) values^70,71^. This study found that the VIF value was not more than 3.3, as shown in Table 2. Therefore, there was no multicollinearity problem.

Discriminant validity was analyzed using the Fornell-lacker test^72^, and Table 3 indicated that this study had a good discriminant validity where the AVEs in each construct were greater than others.

**Table 3:** Results of the Fornell-Larcker test for assessing discriminant validity.

	Anxiety	Work engagement	Facilitating conditions	Innovative behavior	Reward	Self-efficacy	Social influences	TPACK
Anxiety	**0.910**							
Work engagement	− 0.483	**0.889**						
Facilitating conditions	− 0.542	0.848	**0.927**					
Innovative behavior	− 0.460	0.801	0.839	**0.946**				
Reward	− 0.448	0.768	0.821	0.767	**0.867**			
Self-efficacy	− 0.504	0.854	0.847	0.805	0.744	**0.901**		
Social influence	− 0.445	0.813	0.859	0.780	0.794	0.790	**0.862**	
TPACK	− 0.470	0.865	0.841	0.790	0.794	0.839	0.808	**0.897**

All bolded loadings in the diagonal dimension are the square root values of AVE.

### Structural model Evaluation

#### Model fit

Model fit in Smart PLS can be seen from the SRMR, d-ULS, and d_G values ^73^. The difference that exists between the observed correlation and the matric model can be seen in the SRMR value. A good SRMR value is less than 0.08 and this study has 0.04 (Table 4). Furthermore, the difference in the covariance matrix and the empirical covariance matrix can be observed in d-Uls and d_G, which are listed using the composite factor model. In conclusion, this study meets the requirements of a good fit model.

**Table 4:** Results of model fit.

	Saturated model
SRMR	0.044
d_ULS	0.636
d_G	0.857

### Structural model

The structural model was evaluated by examining the significance of the path coefficients using the bootstrapping technique with 5000 resamples ^74,75^. The hypothesis was tested using tailed testing because the type of testing was the directional method. In addition, the complete structural model can be seen in Table 5. Social influence was found to have a significant effect on TPACK knowledge (H1: β = 0.480, *p* < 0.001), work engagement (H2: β = 0.550, *p* < 0.001), self-efficacy (H3: β = 0.537, *p* < 0.001), anxiety (H4: β = − 0.242, *p* < 0.05). The reward had a significant effect on TPACK knowledge (H6: β = 0.414, *p* < 0.001), work engagement (H7: β = 0.332, *p* < 0.001), self-efficacy (H8: β = 0.318, *p* < 0.001), anxiety (H9: β = − 0.225, *p* < 0.05). Meanwhile, facilitating conditions significantly affect mathematics teachers' innovative behavior (H11: β = 0.332, *p* < 0.05). Social influence, TPACK knowledge, anxiety, and work engagement did not significantly affect mathematics teachers' innovative behavior. Self-efficacy also affected mathematics teachers' innovative behavior significantly (H14: β = 0.207, *p* < 0.05).

**Table 5:** Results of the initial hypothesis test.

Direct effect	β	M	STDEV	T Statistics	*P* values
Anxiety—> Innovative behavior	0.010	0.003	0.049	0.197	0.844
Work engagement—> Innovative behavior	0.127	0.116	0.121	1.055	0.292
Facilitating conditions—> Innovative behavior	0.332	0.329	0.146	2.274	0.023
Reward—> Anxiety	− 0.255	− 0.253	0.125	2.043	0.042
Reward—> Work engagement	0.332	0.328	0.080	4.150	0.000
Reward—> Innovative behavior	0.141	0.135	0.101	1.406	0.142
Reward—> Self efficacy	0.318	0.310	0.094	3.379	0.001
Reward—> TPACK	0.414	0.409	0.068	6.084	0.000
Self-efficacy—> Innovative behavior	0.207	0.205	0.103	2.003	0.046
Social influences—> Anxiety	− 0.242	− 0.245	0.106	2.293	0.022
Social influences—> Work engagement	0.550	0.555	0.077	7.152	0.000
Social influences—> Innovative behavior	0.069	0.067	0.100	0.685	0.481
Social influences—> Self efficacy	0.537	0.548	0.099	5.417	0.000
Social influences—> TPACK	0.480	0.486	0.067	7.196	0.000
TPACK—> Innovative behavior	0.064	0.072	0.128	0.497	0.620

While exploring the indirect effects within our analysis, particularly as detailed in Table 6, The analysis indicates that Social Influence significantly affects Innovative Behavior through Self Efficacy, with a relatively high t-statistic and the lowest *p*-value among the paths evaluated.

**Table 6:** indirect effect on Innovative behavior.

Indirect Effect	Original Sample (O)	Sample Mean (M)	Standard Deviation (STDEV)	T Statistics (|O/STDEV|)	*P* Values
Reward—> Anxiety—> Innovative behavior	− 0.002	− 0.000	0.014	0.175	0.861
Social influence—> Anxiety—> Innovative behavior	− 0.002	− 0.001	0.012	0.195	0.846
Reward—> Self efficacy—> Innovative behavior	0.066	0.065	0.041	1.605	0.109
Social influence—> Self Efficacy—> Innovative behavior	0.111	0.104	0.059	1.899	0.058
Reward—> TPACK—> Innovative behavior	0.026	0.033	0.052	0.504	0.614
Social influence—> TPACK—> Innovative behavior	0.030	0.035	0.059	0.513	0.608
Reward—> Work engagement—> Innovative behavior	0.042	0.037	0.041	1.039	0.299
Social influence—> Work engagement—> Innovative behavior	0.070	0.058	0.064	1.088	0.277

**Figure 2:**
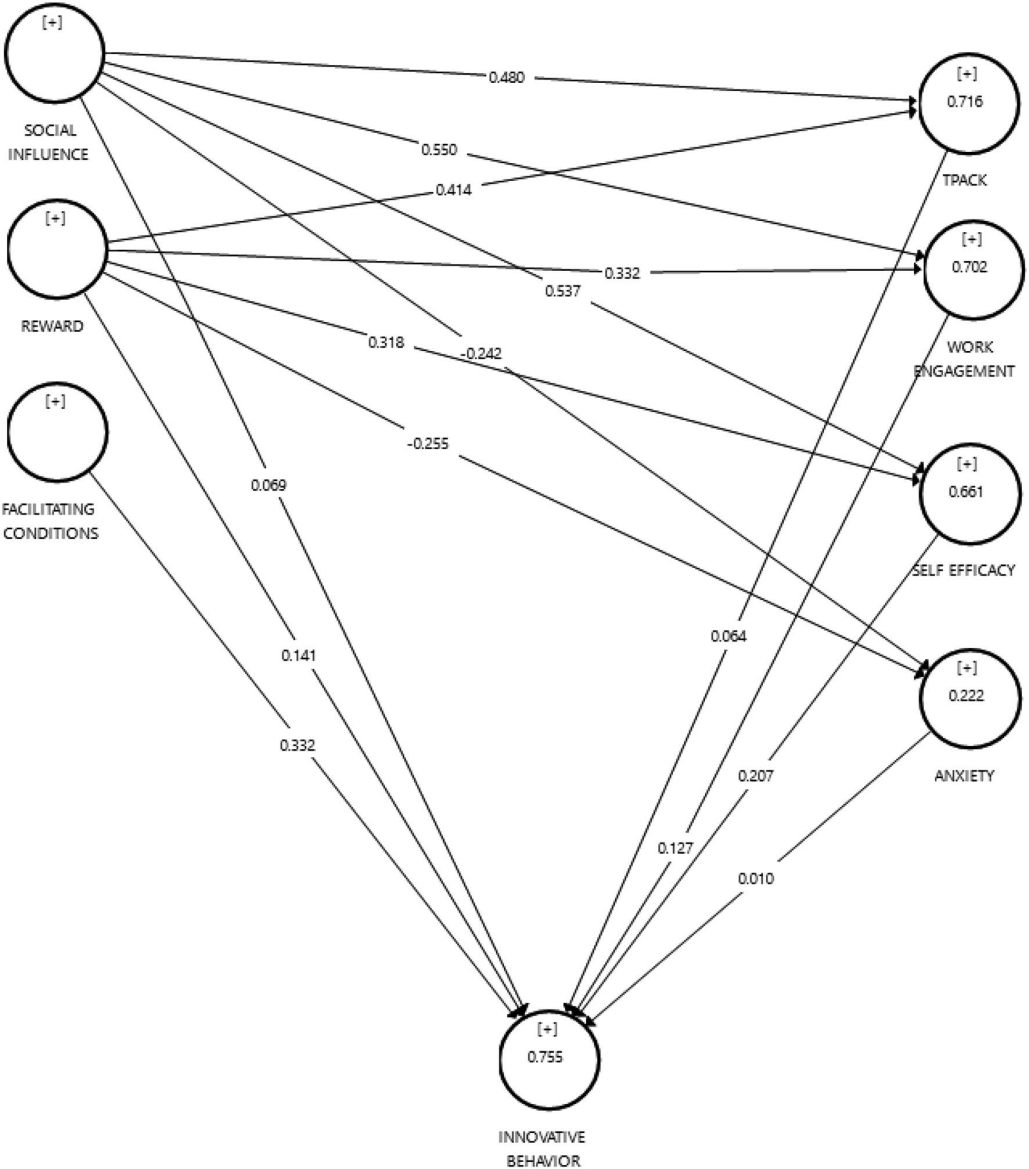
Final model with R^2^ value and path coefficients (β).

Figure 2 showed the P-value and explanatory power (R^2^). The model explained most of the variance in all endogent models such as TPACK knowledge (71.6%), work engagement (70.2%), self-efficacy (66.1%), anxiety (22.2%) and mathematics teacher innovative behavior (75.5%). It had a strong explanation model for the existing available variables. Moreover, the model was proven to have stability and robustness. The significance of the path can be seen in Fig. 2.

## Discussion

This study develops and tests a model to predict factors affecting mathematics teachers' innovative behavior. The major contribution is to modify Social cognitive theory with variables that have a strong relationship with Elementary Mathematics Teachers' Innovative Behavior. From the results of data processing obtained from respondents, this study has empirical findings such as:

Empirical tests reported 10 out of 15 initial hypotheses to be significant. Facilitating conditions and self-efficacy were found to have significant direct effects on elementary mathematics teachers’ innovative behavior.

Interesting findings are facilitating conditions found as a predictor with the first largest effect on mathematics teachers' innovative behavior. This differs from the previous results, where information literacy is the biggest factor affecting innovative behavior^20^. Therefore, elementary mathematics teachers in schools need complete facilities to enhance innovative learning. Respondents were almost 50% of teachers working in rural area schools. In the context of education in rural areas in China, a notable issue persists where numerous classrooms lack adequate facilities, compelling teachers to resort to traditional learning methods. This poses challenges when attempting to introduce innovative approaches to education. Mathematics teachers, in particular, may perceive that having complete and sufficient facilities enhances their effectiveness in implementing novel teaching techniques within the classroom. Moreover, favorable facilitating conditions can also bolster their confidence in making significant advancements in the instructional models employed to teach mathematical concepts. Consequently, the identification of the concept as the primary factor exerting the most substantial influence carries important implications. Schools and government can investigate further what teachers need to support their innovative behavior. Subsequently, providing facilities such as technology-based learning media and full classrooms with technology-based facilities may change and modify teaching methods. Providing training and guidance to mathematics teachers on improving a teacher's innovative behavior might be considered.

The study revealed that direct social influences do not significantly impact the innovative behaviors of mathematics teachers. However, it was found that these social influences have a substantial indirect effect on such behaviors by enhancing teachers' self-efficacy. This finding is consistent with prior research, which also identified only indirect effects of social influences on the variable of innovative behavior^20^. In the specific cultural context of China, where interpersonal relationships are highly valued^12^, the advocacy for innovative teaching methods by respected individuals exerts a notable influence on mathematics educators. This motivates them to explore and adopt novel pedagogical approaches. This discovery is of great practical significance, underscoring the crucial roles that schools, teachers, and governmental entities play in fostering and supporting innovation within the realm of mathematics education.

The unique finding is that rewards significantly affect mathematics teachers' innovative behavior. Teachers in China often have high pressure, chasing learning materials to be mastered by students^76–78^. This may reduce mathematics teachers' innovative behavior. Elementary Mathematics Teachers assert that incentives such as awards or recognition from schools exert a significant impact on their motivation to innovate teaching methods. This finding provides valuable information for schools and decision-makers to reward and recognize teachers with the courage to innovate in classroom teaching and learning activities. In addition, the learning innovation competition may be one of the facilities to reward elementary mathematics teachers who have dared to innovate the learning models used in the classroom.

Based on the Social cognitive theory^36^, self-efficacy has a significant effect on mathematics teachers' innovative behavior. This is appropriate to previous studies where self-efficacy has a strong effect on teacher behavior^8,79^. Individuals with high self-efficacy may can do better than they think. Reinforcement of the concept is one of the right ways for elementary mathematics teachers to innovative behavior. Schools and teachers can pay attention to these aspects.

Meanwhile, elementary mathematics teachers do not consider that TPACK knowledge can significantly encourage innovative behavior. Even though mathematics teachers master TPACK knowledge, it is very difficult to innovate learning without adequate condition facilities and support from the people. Achievement of the goal to enhance the innovative behavior of elementary mathematics teachers can only be realized when the environment aligns with its objectives and provides mutual support to one another.

Anxiety has absolutely no relationship with mathematics teachers' innovative behavior. This interpretation holds on the condition that the environment extends its support, adequate facilities are accessible, the mathematics teachers possess robust self-efficacy to foster educational innovations, and they are unburdened by anxieties when implementing novel teaching and learning practices in the classroom.

## Conclusion

In conclusion, when teachers' innovative behavior is one of the aspects to be improved in the twenty-first century, this study provides empirical evidence by investigating the factors with a significant effect and finding the most influential factors on elementary mathematics teachers' innovative behavior. These results found that facilitating conditions and self-efficacy significantly affect elementary mathematics teachers innovative behavior. Meanwhile, facilitating conditions are the most significant factor affecting mathematics teachers' innovative behavior. Social Influence significantly affects Innovative Behavior through Self Efficacy, as indicated by its *p*-value below 0.1, representing the most substantial indirect effect with the highest t-statistic and lowest *p*-value among the evaluated paths. This study contributes and can be used according to the gap in the innovative behavior of elementary mathematics teachers. Schools and decision-makers can also use the results to improve mathematics teachers' innovative behavior in their respective schools.

## Contribution and implications

The findings contribute theoretically and practically to the study of innovative behavior. Theoretically, the results add to the literature related to the innovative behavior of mathematics teachers at the elementary school level, where instructional innovation is crucial and has a positive impact on students' abilities. It explores the key to success to improve elementary mathematics teachers innovative behavior based on social cognitive theory when mathematics teachers innovative behavior is needed and highlighted at this time. Based on the literature review, study on innovative behavior is very limited, specifically in the context of mathematics teachers. This study provides new knowledge where facilitating conditions and self-efficacy are significant factors for elementary mathematics teachers innovative behavior.

Besides offering theoretical implications, this study also presents practical applications for educational institutions. It sheds light on the determinants of innovative behavior among mathematics teachers, thereby enabling decision-makers and school principals to gain a deeper understanding, offer informed feedback, and develop strategies to foster instructional innovation. Additionally, this research can serve as a valuable resource for local and national education authorities in the development, modification, and refinement of teacher training programs.

## Limitations

Even though this study provides new knowledge, several limitations need to be considered. First, the respondents are small and limited to teachers at the elementary school level hence generalizing the findings and model should be carried out carefully. This study supports future analyses to retest the result with a larger sample and at different levels, such as secondary school or university. Second, it uses a qualitative approach needed for more objective results and in-depth discussion. Third, certain potential predictors, such as teachers' literacy skills, wellbeing, and other relevant factors, could be incorporated and re-evaluated to establish an improved model with enhanced explanatory power. This study believes that innovative behavior is closely related to individual psychology. Therefore, experts in the field of psychology can continue this study.

## Data Availability

The raw data supporting the conclusions of this research will be made available upon request by the author of this publication.

## Appendix

Detail questionnaires

**Table:**

Variable	English version
Facilitating conditions	Schools provide facilities that support teachers to innovate in math learning
	The government and schools often hold training on innovations in math learning
	I can readily access curriculum resources focused on innovative approaches to math learning
Social influences	When I have difficulties innovating in math learning, other math teachers are ready to help
	The school supports teachers to innovate in math learning
	People around me believe that I can innovate in math learning
Reward	The school gives rewards to teachers who can innovate in math learning
	My math learning innovations are appreciated by others
	I am very happy that the school gives appreciation and gifts when I successfully innovate in mathematics learning
TPACK	I have the mathematical knowledge, pedagogical knowledge and technological knowledge to innovate in mathematics
	I can choose new learning media and learning tools that are suitable for the mathematics topic I am teaching
	I can combine technology-based learning media and learning methods to teach mathematics
Work engagement	I am very serious about innovating my way of teaching mathematics
	I am willing to sacrifice my time to innovate new ways of teaching math
	I am always hungry to learn new knowledge, new learning models, and new learning media therefore I can innovate when teaching math
Self efficacy	I am confident that my learning innovations can effectively improve my students' skills
	I believe I can innovate my teaching methods to achieve learning objectives
	I am confident that my students will like my math learning innovations
Anxiety	I am anxious when I have to make innovations in math learning
	I am afraid that my math learning innovations will not be successful
	I am afraid that math learning innovations are a waste of time
Innovative behavior	I often innovate my math learning by using ICT
	I like to use new methods and learning models in math lessons
